# Summer Watering Patterns of Mule Deer in the Great Basin Desert, USA: Implications of Differential Use by Individuals and the Sexes for Management of Water Resources

**DOI:** 10.1100/2012/846218

**Published:** 2012-10-17

**Authors:** Andrew V. Shields, Randy T. Larsen, Jericho C. Whiting

**Affiliations:** ^1^Department of Plant and Wildlife Sciences, Brigham Young University, 275 WIDB, Provo, UT 84602, USA; ^2^Department of Plant and Wildlife Sciences and the Monte L. Bean Life Science Museum, Brigham Young University, 407 WIDB, Provo, UT 84602, USA; ^3^Gonzales-Stoller Surveillance, 120 Technology Drive, Idaho Falls, ID 83401, USA

## Abstract

Changes in the abundance and distribution of free water can negatively influence wildlife in arid regions. Free water is considered a limiting factor for mule deer (*Odocoileus hemionus*) in the Great Basin Desert. Consequently, a better understanding of differential use of water by individuals and the sexes could influence the conservation and management of mule deer and water resources in their habitats. We deployed remote cameras at all known water sources (13 wildlife water developments and 4 springs) on one mountain range in western Utah, USA, during summer from 2007 to 2011 to document frequency and timing of water use, number of water sources used by males and females, and to estimate population size from individually identified mule deer. Male and female mule deer used different water sources but visited that resource at similar frequencies. Individual mule deer used few water sources and exhibited high fidelity to that resource. Wildlife water developments were frequently used by both sexes. Our results highlight the differing use of water sources by sexes and individual mule deer. This information will help guide managers when siting and reprovisioning wildlife water developments meant to benefit mule deer and will contribute to the conservation and management of this species.

## 1. Introduction

Free water is a critical resource for humans and wildlife in arid regions of the world. Several factors, however, influence both the current and future availability of water in these regions. Growing human populations have increased the need for water globally [1]. Similarly, climate change will affect the quantity and distribution of water available to humans and wildlife [2, 3]. Loss and degradation of natural water sources in the arid western USA has occurred and likely will continue given ongoing and projected anthropogenic influences in that area [4–7]. This change in abundance and distribution of water can negatively influence populations of wildlife and has created a conflict between the needs of humans and wildlife for water resources. For example, in Joshua Tree National Park (JTNP), USA, the number of natural, perennial water sources declined from 19 in the 1950s to 5 in 2004, partly because of anthropogenic use of surface and ground water [8]. This reduction in water sources subsequently decreased critical summer habitat for bighorn sheep (*Ovis canadensis*) [8]. This reduction of summer habitat for bighorns in JTNP, however, was partially mitigated by construction of wildlife water developments [8].

**Figure 1:**
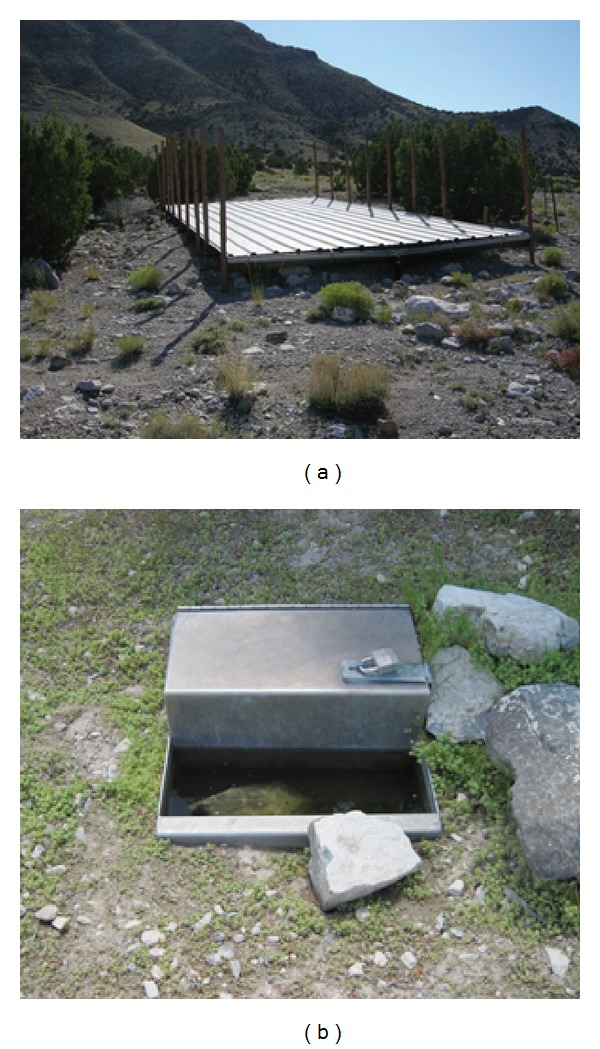
Typical wildlife water development on the Thomas-Dugway Mountains in western Utah, USA, including catchment apron (a) and drinker (b) where we photographed mule deer using water sources, 2007–2011.

Constructing water developments is one way to mitigate the current and projected loss of natural water sources used by wildlife in arid regions. Indeed, since the 1940s, nearly 7000 of these devices have been built in the western USA with more than $1,000,000 in combined annual expenditures [7, 9]. Several variations of wildlife water developments exist, but their primary function is to catch and store precipitation, which is then made accessible to wildlife during dry periods [10] (Figure 1). Construction of wildlife water developments, however, has become controversial, particularly in the southwestern USA. Concerns exist about the efficacy of water developments [11–14], the compatibility of these developments with wilderness values [5, 15], and the potential for negative effects from these devices (i.e., increased predation, competition, disease transmission [9, 11], and negative ecosystem interactions between native and exotic species around these locations [13]). Conflict over wildlife water developments has persisted for nearly 20 years, and even the decision processes, civility, and human dignity associated with constructing these devices have been criticized [7, 16]. This controversy has prompted numerous studies to expand our understanding of the ecological and biological effects of providing human-built water sources for wildlife in arid and semiarid environments [5]. 

In the Great Basin Desert, water availability is suspected to be a limiting factor for mule deer (*Odocoileus hemionus*) [17], particularly during summer; however, little research has been conducted on this topic. Mule deer are a popular game species [17], an important prey item for carnivores [18, 19], and an integral part of the ecosystems of the western USA [20]. Indeed, these deer have been implicated as a central component of a potential disequilibrium of predators and prey in the Great Basin, which ostensibly has affected ecosystem and community dynamics across this region [18]. In much of the Great Basin, wildlife water developments have been built to mitigate scarcity of water and benefit mule deer populations. However, the extent to which additional water provided by water developments benefits these ungulates remains unclear [5, 21]. 

Moreover, very little research has been conducted on long-term use of water sources by mule deer in the Great Basin Desert, and a gap in knowledge exists regarding how sexes of these deer use water differently. Recent research on water use by bighorn sheep, for example, highlighted the need to understand how males and females use water differently in order to effectively conserve and manage these specialized ungulates and their habitats [3]. We used remote cameras to document use of 17 water sources (13 wildlife water developments and 4 natural springs) across five years during summer by individually identified mule deer in the Great Basin, USA. We determined frequency and timing of visits to water by mule deer, evaluated differences in the use of that resource between sexes and by year, and identified the number of water sources used by males and females. We also estimated population abundance using photographs of identifiable individuals visiting water sources. General information about how mule deer use water, including wildlife water developments, may help alleviate some of the conflict surrounding the loss of natural water sources. Further, information about how the sexes use water differently in arid ecosystems will aid the development of management and conservation strategies to ensure the long-term persistence of this species over the coming decades during a projected global water shortage. 

## 2. Materials and Methods

### 2.1. Study Area

We quantified use of 17 water sources (13 wildlife water developments and 4 springs) by mule deer on the Thomas-Dugway Mountains in western Utah. Those 17 water sources represented all known wildlife water developments and springs accessible to ungulates on the Thomas-Dugway range based on current and historical maps and other research regarding water use by chukars (*Alectoris chukar*) in that area [22, 23]. This mountain range is located in Juab and Tooele counties (N 39°51′33′′, W 113°5′29′′) within the Great Basin Desert. As with other mountain ranges in the Great Basin, the Thomas-Dugway range extends in a north-south direction and is approximately 40 km long and 13 km wide. Elevations range from 1380 to 2135 m. Average annual precipitation over a thirty year period (1981–2010) for this area was 224.8 mm with only 45.0 mm occurring in summer (June–August). Summer high and low temperatures for the same time period averaged 33.5°C and 13.7°C (Table 1). Autumn (September–November) high and low temperatures were cooler and averaged 19.6°C and 1.7°C, with an average precipitation of 55.4 mm. Winter (December–February) high and low average temperatures were 5.5°C and −1.5°C, with an average of 47.5 mm of precipitation, largely as snow. Average spring (March–May) high and low temperatures were 18.8°C and 1.8°C, and average spring precipitation was 76.9 mm. The study area was hotter and drier than average during the initial years, then became cooler and wetter during later years (Table 1) (Western Regional Climate Center, http://www.wrcc.dri.edu/). Major land-cover types and vegetation communities on the Thomas-Dugway Mountains included the following: Great Basin pinyon (*Pinus* spp.)-juniper (*Juniperus* spp.) woodland, Great Basin xeric-mixed sagebrush (*Artemisia* spp.) shrubland, intermountain basins semidesert shrub steppe, inter-mountain basins mixed salt desert scrub, invasive annual grassland, and intermountain basins cliff and canyon [24].

Water sources on the Thomas-Dugway Mountains occurred at varying elevations between 1500 and 1950 m and in several different vegetation types. Wildlife water developments were located in a variety of habitats, but generally occurred in washes or on small ridges at the base of the mountain range (1561–1772 m) (Figure 1). All wildlife water developments we evaluated were constructed specifically for ungulate use and were within areas used by mule deer. Springs were also located in a diversity of habitats with three near the base of the mountains and one on a primary ridge (1318–1918 m). Average (*± *SD) distance from one water source to the next nearest water source was 3.3 km (±1.4, range = 1.8 to 6.3). All water sources held water during the study period with the exception of one wildlife water development that sporadically malfunctioned during each year. Only one of the 17 water sources was fenced [25]. 

### 2.2. Sampling

We placed passive infra-red (PIR) cameras (The Digital 3.2, Camtrakker Inc, Watkinsville, Georgia; Pixcontroller, universal controller board Sony DSC P-32 camera, Export, Pennsylvania; or PC900, Reconyx Inc, Holmen, Wisconsin) at all known water sources each summer from 2007 to 2011. Our PIR cameras required both heat and motion to activate, and we placed cameras 3-4 m from water sources to detect and photograph animals using that resource. We aimed cameras to detect motion 1 m in height above the water source and visited each water source every 10–14 days throughout the sampling period to replace batteries and memory cards and ensure cameras were functioning properly. To minimize disturbance to mule deer, we visited water sources primarily during daylight hours and typically spent less than 20 minutes at each water source. We assumed mule deer photographed at water sources drank, an assumption validated by O'Brien et al. [26] using remote videography.

We selected a 40-day window from 15 July to 23 August for sampling because use of water sources was minimal before this period (A. Shields, unpublished data). This window also corresponded with the hottest and driest time of the year. Moreover, scars and other pelage irregularities became difficult to see in late August-early September as mule deer hair turned from red to grey. In 2008, cameras were deployed on 15 July but were removed from water sources in late July because several were stolen. During the other years (2007, 2009–2011) cameras were operational throughout the 40-day window with the exception of occasional times when cameras malfunctioned. For our analyses, we excluded one spring because we did not photograph any deer at that location during the study. 

**Figure 2:**
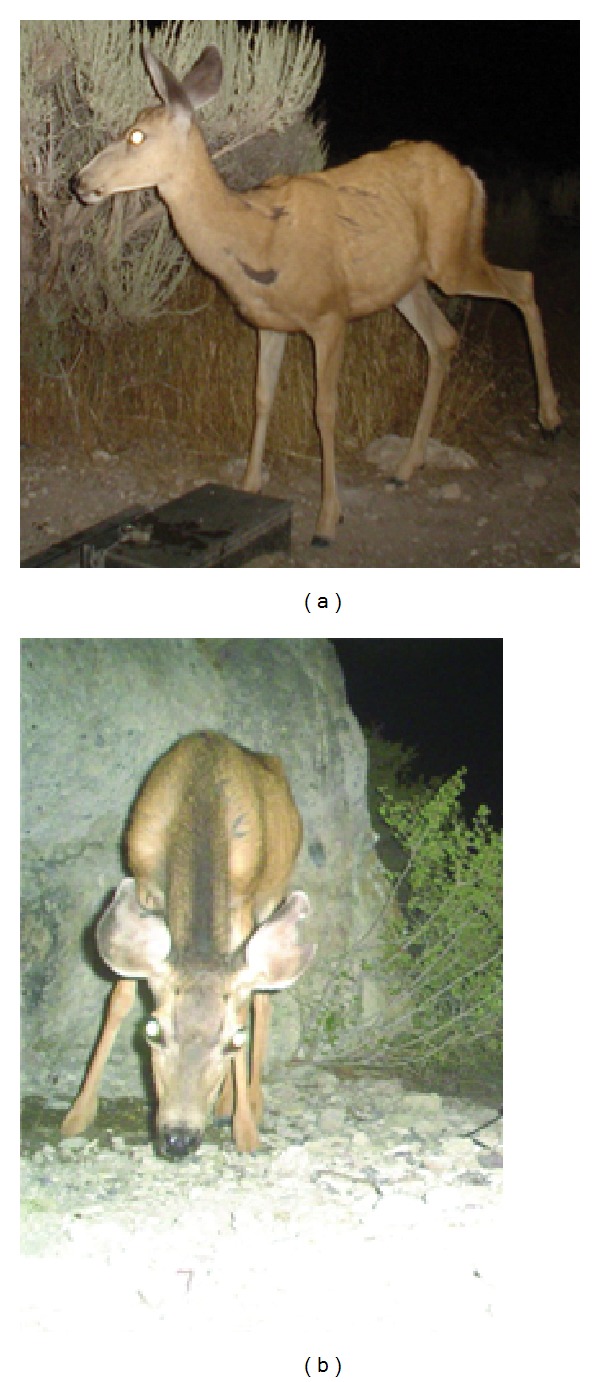
Photographs of female mule deer taken by remote cameras showing distinguishing features including scars (a) and notches in both ears (b) that we used to identify individuals at water sources in Utah, USA, 2007–2011.

Once photographs were collected, we identified individual mule deer based on antler characteristics (males), pelage irregularities (e.g., scars and cuts on males and females), and other distinguishing marks [27]. For example, one female was missing an eye, another had several cuts in one ear, and a third was missing a large piece from one ear (Figure 2). We assigned each deer a unique identifier, grouped all photographs of an individual taken throughout the summer sampling period, and repeated this process for each identifiable deer. Some female deer did not have clearly distinguishable marks, so we excluded photographs of those deer from our analyses of frequency, timing, and number of water sources used. We were able to identify a few individuals visiting water sources across years; however, we recorded them as separate deer each year because of the difficulty in identifying most individuals across years (different scars, different antler configurations, etc.). 

### 2.3. Water Source Use

We extracted date and time from each photograph and standardized them to a Julian date. To determine frequency of visits by an individual mule deer to a water source, we calculated the difference in time between the first photograph of that individual at a water source and the last photograph of the previous visit to a water source. Occasionally cameras malfunctioned, and we did not use the elapsed times between visits to water when this occurred. We classified a visit as a photograph or series of photographs preceded by at least a 25-minute lapse of time since the last photograph of a mule deer [28]. We calculated the mean elapsed time between visits to water for each identifiable deer within a year and used these values for further analysis. We used analysis of variance (ANOVA) to assess differences between years and sexes and the sex by year interaction in mean hours between visits. Following a significant ANOVA result (*P* < 0.05), we conducted post-hoc tests (Tukey's adjustment for multiplicity of tests, *T*) to investigate differences by year, sex, and the interaction of those variables.

To determine diel timing of water source use, we recorded the time of the first photograph of each visit by an identified deer to a water source. To test for differences between sexes and years, we used a MANOVA test. Because time of day was a circular variable, we used the sine and cosine function to transform this variable for analyses and used these transformed values as the two response variables [29]. To determine differential water source use by sexes, we recorded the number of times identified deer used each water source. We combined all years and plotted these data as proportion of visits by each sex. We determined that a water source was used primarily by one sex if >75% of the combined events occurred by one sex at that water source. For each identified deer, we recorded the number of water sources used and the number of times each deer changed water sources. To test for differences between sexes and years for those variables, we used an ANOVA test. We also calculated the minimum distance traveled by deer that changed water sources at least once by summing the distances of all known movements by an individual between water sources. We evaluated assumptions (e.g., normality, homogeneity of variance) of the ANOVA and MANOVA tests graphically and used Program R to conduct all statistical tests [30]. 

### 2.4. Abundance Estimation

Using photographs of individually recognized mule deer at water sources, we estimated abundance of females using the Poisson log-normal mixed-effects mark-resight model [31] in program MARK [32]. We only generated abundance estimates of females because we could identify all males and did not have unidentified photographs of any males. For females, the method we used is a relatively new mark-resight model that allows for the estimation of the number of unmarked individuals in the population and derives an estimate of abundance and mean resighting rate from the total number of marked and unmarked animals resighted [33, 34]. Rather than marking individuals, we considered deer that we had individually identified based on scars and pelage irregularities as marked. We used one sampling interval of 14 days (from 9 August to 23 August in 2007 and 2009 to 2011 and 16 July to 30 July in 2008) and sampled with replacement, where an individual was counted as resighted each time it visited a water source. Sampling dates were different in 2008 because cameras were stolen and we stopped sampling at the end of July. Some identifiable deer were not detected at water sources during resight sampling so we only considered deer marked if they were individually identifiable and photographed during the week prior to or during the 14-day sampling interval. To compensate for differences in the number of photographs of an individual per visit (longer visit = more photos), we treated each visit, rather than each photograph, as a resight regardless of how many photographs were collected of an individual during that visit. 

## 3. Results

### 3.1. Water Source Use

We sampled 2193 camera days (camera active 24 hours at a water source) from 2007 to 2011 (mean per year = 439 camera days; range 164 to 572 camera days). We collected a total of 13,686 photographs of mule deer at 16 of the 17 water sources over the five years of sampling (Table 2). From these photographs, we identified 76 males and 116 females and tallied 790 drinking events by males and 1179 drinking events by females from identified individuals during the study period (Table 2). 

**Figure 3:**
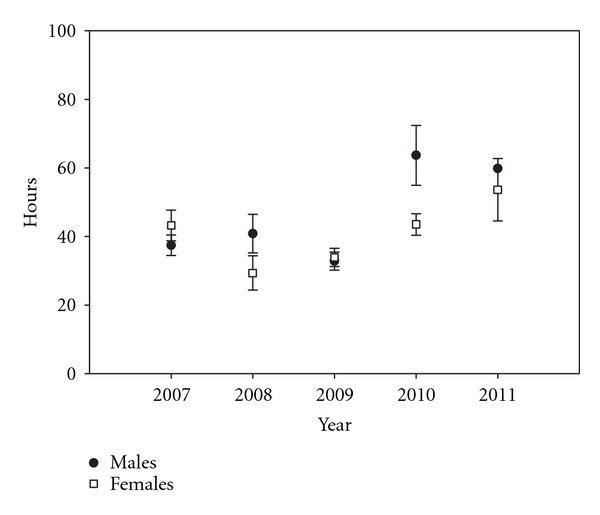
Hours between visits (±95% CIs) to water sources by male and female mule deer during summer (July and August) in western Utah, USA, 2007–2011. The confidence interval is missing for males in 2011, because there were too few visits to water by males in that year to generate a meaningful interval.

Mean number of hours (± SD) between visits for males was 38.2 (±26.2; median = 29.8) and 39.9 (±29.2; median = 30.9) for females across all five summers. Males visited water most frequently in 2009 (once every 32.8 ± 21.9 hours; median = 25.7) and least frequently in 2010 (once every 63.6 ± 33.0 hours; median = 66.0) (Figure 3). Females visited water sources most frequently in 2008 (once every 29.3 ± 21.4 hours; median = 31.5) and least frequently in 2011 (once every 53.6 ± 37.1 hours; median = 43.3) (Figure 3). Frequency of visits to water by males and females combined varied between years (*F* = 6.25,  *df* = 4, 158, *P* < 0.01). Post hoc means comparisons indicated low frequency of visits in 2010 and 2011 compared to early years. Significant differences existed for both sexes combined in 2007 (*T *= 12.96, *P* = 0.04), 2008 (*T *= 15.82, *P* = 0.02), and 2009 (*T *= 20.09, *P* < 0.01) compared with 2010, as well as in 2009 compared with 2011 (*T *= 18.52, *P* < 0.01). Combining all years, frequency of watering did not differ between sexes (*F* = 0.20,  *df* = 1, 161, *P* = 0.66); however, there was a significant interaction with sex and year. Frequency of visits to water did not differ across years for females. During 2007 (*T* = 33.89, *P* < 0.01), 2008 (*T *= 25.84, *P* = 0.05), and 2009 (*T *= 36.43, *P* < 0.01), males visited water more frequently than in 2010 (*F* = 3.97, *df* = 4, 153, *P* < 0.01). 

**Figure 4:**
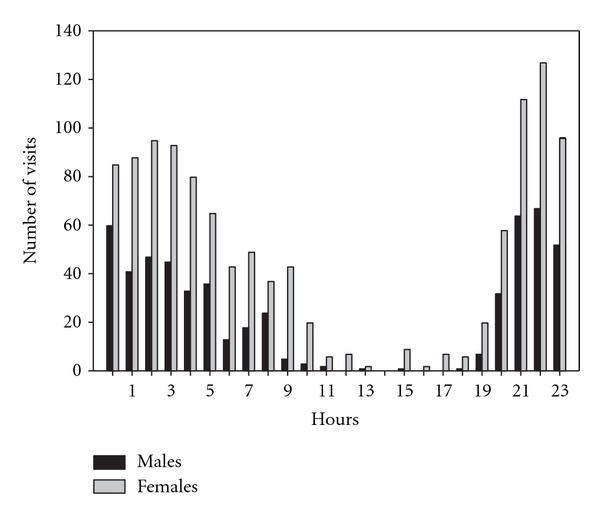
Timing of visits to water sources by identified male and female mule deer during summer (July and August) in western Utah, USA, 2007–2011.

Mule deer visited water infrequently from 1100 hours to 1900 hours (3% of all visits, Figure 4). Both males and females visited water more often during the evening than morning. The most visits in any hour were recorded from 2200–2300 hours for both males (12% of all male visits) and females (11% of all female visits) (Figure 4). Most visits to water sources occurred at night, with 81% of male and 73% of female visits occurring between 2100 and 0600 hours. There was a difference in timing of visits between sexes across years (*F* = 16.92, *df* = 1, 1967, *P* < 0.01); however, general patterns of use were similar (Figure 4). There was also a difference in timing of visits between years for both sexes combined (*F *= 2.99, *df* = 4, 1964, *P* < 0.01).

**Figure 5:**
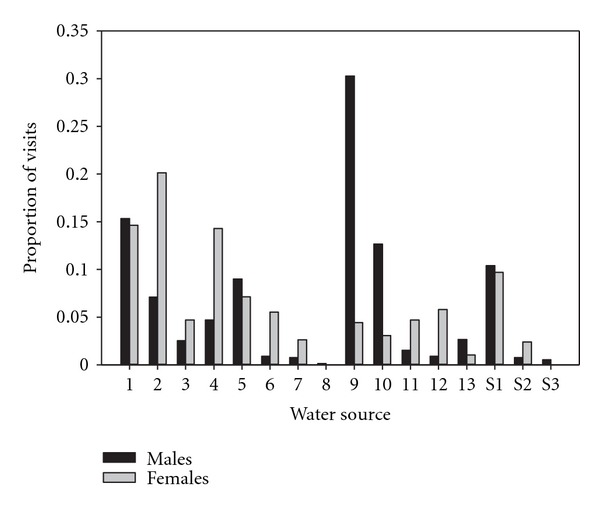
Proportion of visits by male and female mule deer to water sources in western Utah, USA, 2007–2011. Water sources 1 to 13 were wildlife water developments; S1 to S3 were natural and modified springs.

Although 14 water sources were used by males and females, 2 water sources (9 and 10) were used primarily by males and 6 sources (4, 6, 7, 11, 12, and S2) were used predominately by females (Figure 5). Four water sources (1, 2, 4, and S1) received 59% of female visits, whereas nine water sources each received <5% of female visits, including two water sources that were not used by females. Four water sources (1, 9, 10, and S1) received 69% of male visits, whereas ten water sources each received <5% of male visits. Wildlife water developments were used extensively with 90% of male visits and 88% of female visits to those water sources. 

Individual males used an average of 1.5 (SD ± 0.8, range = 1 to 5) water sources each year, whereas individual females used 1.3 (SD ± 0.7, range = 1 to 5) water sources per year. Males changed water sources an average of 1.3 (SD ± 2.2, range = 0 to 8) times each summer and traveled an average minimum distance of 11 km (SD ± 7.8, range = 2.7 to 25.7, *n* = 28) across changes. Females changed water sources an average of 0.7 (SD ± 1.8, range = 0 to 12) times and traveled a minimum average distance of 13.4 km (SD ± 12, range = 1.7 to 38.7, *n* = 20) across changes. 

### 3.2. Abundance Estimation

 We included 109 female deer from all years in our model of abundance as individually identifiable and obtained 598 resightings of those individuals (Table 3). We also tallied 362 total visits by unmarked females. The Poisson-log normal model produced reasonable estimates of abundance of females using these marked individuals (Table 3). Abundance estimates indicated that number of female mule deer in our study area was stable from 2007 to 2011 (Table 3). 

## 4. Discussion

In our study, most water sources were used by both sexes; however, two (12.5%) wildlife water developments were used primarily by males and six (37.5%) different wildlife water developments were used predominately by females. Those patterns of water use by the sexes were consistent across the five years of our study, indicating high fidelity by the sexes for specific water sources. Recent research indicated that although home ranges overlapped considerably for male and female bighorn sheep, use of different water sources occurred and that consideration should be given to the separate habitat requirements for each sex when evaluating the use of water [3]. Additionally, wildlife water developments constructed in areas used by one sex may not be beneficial for the other [7, 35, 36]. More work is needed on this topic to determine how habitat and landscape features may influence use of water sources by males and females differently.

Across the five years of our study, individual male and female mule deer used relatively few water sources. A pattern was not evident relating the number of water sources used to differing levels of precipitation across years. In other studies, when water catchments were closed, thereby eliminating availability of this resource, mule deer females traveled outside their home range to find other water sources [37]. In our study area, all water sources remained available to deer across the five years, and although deer used as many as five water sources, 81% of females and 63% of males were photographed at only one water source. Similarly, a mature male that we were able to identify (based on a tear in his right ear) used the same water source exclusively each year from 2007 to 2010. These results indicate that in our study area individual mule deer exhibit high fidelity to water sources both within and across years. Current research is stressing the importance of the variability in individual behavior of wildlife with regards to conservation and management of species [38, 39]. Thus, loss of natural water sources may affect certain individuals and not others; and the siting, reprovisioning, and building of water developments for mule deer may benefit only certain individuals. 

Researchers have stressed the importance of documenting water use by wildlife covering multiple years and wet-dry periods [5, 9, 21]. Our study covered five years comprising dry (2007–2009) and wet (2010-2011) periods. The frequency of water use generally followed these weather patterns. Low precipitation and high temperatures early in the study period corresponded with greater frequency of use, and high precipitation and lower temperatures later in the study period corresponded with lower frequency of water use (Figure 3, Table 1). These results are consistent with mule deer studies in other areas [37, 40] and other ungulate studies in the Great Basin [41]. Spring 2011 was much wetter than normal (196% of 30 year mean, Table 1), and thus, mule deer use of water sources during summer 2011 was much lower than other years. Indeed, males only visited water sources on ten occasions; whereas, females visited this resource on 104 occasions. We hypothesize that frequency of water use was influenced by the amount of moisture available in forage and availability of water in temporary sources (e.g., puddles). Availability of water in forage is further influenced by evapotranspiration rates which are correlated with humidity and temperature. We also hypothesize that females visited water sources more frequently than males during summer because of lactation demands [42, 43]. 

Mule deer visited water sources primarily from late evening until early morning with very few visits recorded during the middle part of the day. Other studies showed peak visitation in the evening and a marked decrease in visits through the night and morning for females in Arizona [37] and males and females in California [44]. Our results indicated highest visitation rates in the evening, but showed continued high visitation through the night and into the morning before visits decreased. Other authors have suggested mule deer use water in the evening in response to dehydration that occurs throughout the day [44] and to restrict movement during the hottest part of the day. Our results are consistent with this hypothesis. 

Consistent use of water sources by mule deer in arid environments can provide opportunities for estimating abundance and thus help determine how human-provided water sources influence wildlife populations. Hervert and Krausman [37] suggested that because female deer are dependent on water sources when temperatures are high, and females visited water sources once per day, censusing female deer at water sources may be possible. Indeed, other researchers have been able to identify individual white-tailed deer using unique antler configurations [27] and several species of felids based on spot patterns in pelage [45–47]. The consistent use of water by mule deer in our study area and the ability to identify those individuals allowed us to estimate abundance noninvasively using mark-resight procedures. These methods can likely be extended to other species, particularly in small, isolated populations where animals consistently visit a particular area, such as water sources in an arid environment. Using these methods may be particularly useful in quantifying a decrease or increase in abundance of animals in relationship to changes in availability of water (e.g., loss of springs or addition of wildlife water developments). 

Water is an important resource for many animals in arid environments [48, 49]. Climate change will affect the future quantity and quality of water for wildlife around the world [2, 3] and in the Great Basin, USA [50]. Human-provided water sources may help to reduce the conflict between the needs of humans and wildlife for water [6, 7]. Knowledge about the use of water sources by the sexes will help managers and scientists develop strategies to conserve and manage species that rely on this resource [3]. Our results indicate that male and female mule deer visited water sources at similar frequencies, but used different water sources. Individual male and female mule deer used relatively few water sources and exhibited high fidelity to this resource both within and across years. Additionally, most wildlife water developments were used extensively by both sexes and may have mitigated the scarcity of naturally occurring free water. Our results highlight the differing use of water sources by sexes and individual mule deer during summer. This information will help guide managers when siting, reprovisioning, and building wildlife water developments meant to benefit male and female mule deer and will contribute to the conservation and management of this species.

## Figures and Tables

**Table 1:** Spring (March to May) and summer (June to August) mean temperature (±SD) and total precipitation in western Utah, USA, 2007–2011 where we evaluated mule deer use of water sources.

Year	Spring		Summer	
	Temperature (°C)	Precipitation (mm)	Temperature (°C)	Precipitation (mm)
2007	10.9 ± 3.7	8.8	25.1 ± 2.3	31.6
2008	8.7 ± 4.1	33.7	24.5 ± 2.5	21.5
2009	10.2 ± 4.8	66.2	22.3 ± 2.6	43.4
2010	8.4 ± 2.5	101.2	24.1 ± 2.2	39.6
2011	8.6 ± 2.4	150.9	23.3 ± 3.1	39.4

30 year average	10.3	76.9	23.6	45

**Table 2:** Number of mule deer photographs taken and identified to individual, as well as the number of unique males and females detected using water sources during summer (July and August) in western Utah, USA, 2007–2011.

Year	Number of photos	Number identified	Percent identified	Number of males identified	Number of females identified
2007	3614	2803	0.78	27	26
2008	1566	829	0.53	19	19
2009	4727	4139	0.88	16	26
2010	2857	2290	0.80	9	21
2011	922	562	0.61	5	24

Total	13,686	10,623	0.78	76	116

**Table 3:** Number of identifiable deer, resightings, visits by unmarked individuals, abundance estimates ($\hat{N}$), and mean resighting rates ($\overset{-}{x}$ RR) along with standard errors (SE) and 95 percent confidence intervals (L95CI and U95CI for lower and upper 95% confidence intervals) for female mule deer in western Utah, USA, 2007–2011 estimated using program MARK.

Year	Identifiable deer	Number of resightings	Unmarked visits	$\hat{N}$	SE	L95CI	U95CI	$\overset{-}{x}$ RR	SE	L95CI	U95CI
2007	20	100	69	34	3.60	26	41	4.99	0.83	3.36	6.63
2008	19	84	142	52	6.49	40	65	4.22	0.60	3.05	5.40
2009	25	188	76	35	2.31	30	39	7.75	1.07	5.66	9.85
2010	21	141	45	28	1.51	25	31	6.60	0.76	5.11	8.09
2011	24	85	32	38	4.27	29	46	2.29	0.42	1.47	3.12
